# Genicular Artery Embolization Using Resorbable Gelatin Microspheres for Refractory Knee Pain: Technique, Safety and Clinical Outcome

**DOI:** 10.1007/s00270-025-04274-6

**Published:** 2025-11-18

**Authors:** A. Taheri Amin, A. Hübner, E. Kemmer, P. Krüselmann, F. Ziayee, L. Wilms, B. Fink, K. Jannusch, NP. Hoff, B. Homey, P. Minko

**Affiliations:** 1 Department of Diagnostic and Interventional Radiology, Charité Universitätsmedizin Berlin, Corporate Member of Freie Universität Berlin and Humboldt-Universität Zu Berlin, Berlin, Germany; 2 Department of Diagnostic and Interventional Radiology, Medical Faculty, University Hospital Duesseldorf, 40225 Duesseldorf, Germany; 3 Department of Dermatology, Medical Faculty, University Hospital Duesseldorf, 40225 Duesseldorf, Germany

## Abstract

**Purpose:**

To evaluate the safety, technique and clinical outcome of genicular artery embolization (GAE) using resorbable gelatin microspheres (RGM) in patients with knee osteoarthritis (OA) or persistent pain after total knee replacement (TKR).

**Material and Methods:**

In this prospective observational study, 45 patients with knee OA (Kellgren–Lawrence 1–4, n = 35) or post-TKR pain (n = 10) were included. GAE was performed using 100–300 µm RGM. Embolic volume and treated vessels were documented. Clinical outcome was evaluated using the Knee Injury and Osteoarthritis Outcome Score (KOOS) and Numeric Rating Scale (NRS) at baseline, 6 weeks, 3 and 6 months. Psychological comorbidities were screened using the Hospital Anxiety and Depression Scale (HADS). Adverse events were recorded.

**Results:**

At 6 months, mean NRS improved by 35% and KOOS subscale pain by 55% (p < 0.001) with no significant differences between OA grades and post-TKR. A median of 3 (2–6) vessels were embolized with a mean total embolic volume of 6.5 mL (2.1–18.0 mL) per session. Patients with a HADS score ≥ 8 showed less improvements in the KOOS (up to 2.2 points less per HADS point increase; p < 0.05). Total embolic volume was significantly higher in advanced OA and post-TKR, averaging at 2.7 mL more per OA grade (p < 0.0001). No major complications were observed.

**Conclusion:**

GAE using RGM in doses higher than 2 mL is safe and demonstrates short-term clinical efficacy across all OA grades and in post-TKR pain. Patients with severe OA and post-TKR pain received higher embolic volumes. A HADS score ≥ 8 is associated with reduced clinical benefit.

**Graphic Abstract:**

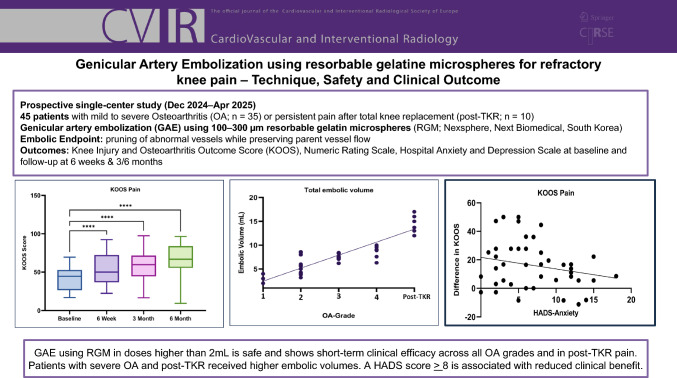

**Supplementary Information:**

The online version contains supplementary material available at 10.1007/s00270-025-04274-6.

## Introduction

Genicular artery embolization (GAE) is increasingly recognized as an established, treatment option for knee pain refractory to conservative treatment, with efficacy confirmed in meta-analyses [1–3]. As the procedure gains popularity, technique and patient selection remain heterogeneous, which contributes to variable outcomes.

A key source of heterogeneity is the choice of embolic agent. Permanent embolics such as microspheres or polyvinyl alcohol achieve durable vessel occlusion but are often associated with transient skin discolorations [4, 5]. Temporary embolics such as imipenem–cilastatin (IPM/CS) or resorbable gelatin microspheres (RGM; Nexsphere, Next Biomedical, South Korea) are resorbed within hours [6]. Their clinical efficacy in mild to moderate osteoarthritis (OA) and safety appear comparable to permanent agents, while causing fewer skin discolorations and theoretically posing less risk in non-target embolization [7–9].

Patient selection is another factor influencing outcomes. Several studies using temporary embolic agents have reported reduced efficacy in patients with advanced OA on knee radiographs and MRI [10–13]. To establish GAE as a standard practice, however, therapeutic strategies must be extended to a broad patient spectrum. This includes patients across all OA grades as well as the 20% of individuals experiencing persistent pain after total knee replacement (post-TKR)[14]. Early reports suggest that advanced OA may require more aggressive embolization with higher embolic volumes [15–17].

The aim of this prospective exploratory study was to evaluate the safety and short-term efficacy of GAE using RGM across different OA grades as well as in post-TKR pain and to identify determinants of treatment success to guide more patient-centered embolization strategies.

## Materials and Methods

### Study Design

This prospective single-center observational study was conducted at the University Hospital of Duesseldorf between December 2024 and April 2025. Eligible participants were 18–90 years old, had radiographic evidence of knee OA or post-TKR and knee pain refractory to ≥ 6 months of conservative treatment. Exclusion criteria were standard contraindications to angiography. In patients with bilateral knee OA, the side with predominant pain was treated first and overall pain was reassessed after six weeks before considering contralateral treatment. Pre-interventional radiographs were reviewed in consensus by two musculoskeletal radiologists (F.Z. and E.K), and OA was graded using the Kellgren–Lawrence scale (K&L).

### Procedure

All interventions were performed by a single IR with more than 15 years of experience (P.M.) to minimize inter-operator variability. Radiographs were reviewed by the IR pre-procedurally. Ipsilateral antegrade transfemoral access was obtained without an introducer sheath to reduce access site diameter [18]. Digital subtraction angiography (DSA) was performed at the mid-third of the distal superficial femoral artery using a 4 F Cobra catheter (Infiniti, Cordis Medical, Austria) and iodinated contrast medium (300 mg/mL Accupaque, GE Healthcare, USA) to delineate vascular anatomy. Superselective catheterization of genicular arteries was achieved with a 1.7F microcatheter (Pursue, Merit Medical, USA). Embolization was performed upon detection of a hyperemic blush using 100–300 µm RGM. RGM were prepared immediately before the first injection according to the manufacturer’s instructions: mixed with 10 mL saline for one minute, hydrated for 30 s, then diluted with 10 mL of the contrast agent mentioned above. Embolization was performed in aliquots to the same subjective endpoint, defined as pruning of abnormal neovessels while preserving parent vessel inflow. Technical success was defined as catheterization of all visible genicular arteries. Procedural parameters including radiations exposure, number of embolized vessels and embolic volume were recorded.

Patients were observed in the outpatient clinic for 4 h before discharge. They were advised to avoid heavy lifting or strenuous physical activity for 24 h but could otherwise resume daily activities. Vascular complications were assessed clinically and by duplex/Doppler ultrasound before discharge and 24-h post-intervention. Skin discolorations were evaluated by clinical inspection and 3D high-resolution imaging (Vectra WB360, Canfield Scientific, USA) before, 24 h and six weeks after embolization, with all images reviewed by a board-certified dermatologist (NP.H.). The system has been validated for objective detection and quantification of skin color changes [19]. All complications were recorded and classified according to the modified CIRSE Standards for Complication Reporting [20].

Pre-interventional assessment included screening for psychological comorbidities using the Hospital Anxiety and Depression Scale (HADS) and documentation of previous pain therapies. Clinical outcomes were evaluated using the Numeric Rating Scale (NRS) and the Knee Injury and Osteoarthritis Outcome Score (KOOS) at baseline, six weeks, three and six months. At baseline, the questionnaire was explained in person and completed independently by the patients. Follow-up assessments were conducted by mail, with telephone support available if needed. Clinical success was defined as improvement of ≥ 10 points in KOOS at any follow-up compared to baseline, consistent with ongoing randomized controlled trial protocols on GAE [21, 22].

## Statistical Analysis

This observational study had an exploratory design. Thus, no sample size calculation was performed. The target sample was determined by consecutive recruitment during the study period.

Normality of variables was assessed using the Shapiro–Wilk test. Data are expressed as mean ± SD for normally distributed variables and as median (range) for non-normally distributed variables. Repeated-measures ANOVA with post hoc multiple comparisons (Bonferroni correction) was used to assess changes in NRS and KOOS scores between baseline and each follow-up.

Patients were stratified into subgroups according to radiographic OA severity (K&L 1–4/post-TKR), HADS subscales (anxiety/depression ≥ 8) and BMI score (< 25 kg/m^2^ or ≥ 25 kg/m^2^). Between-group differences in KOOS change (baseline vs. six months) and embolic volume were analyzed with the Mann–Whitney U or Kruskal–Wallis test as appropriate. When significant differences were detected, linear regression estimated the effect per unit increase in predictor (HADS score, OA grade). Correlations were further explored with Spearman’s rank coefficient (*ρ*). Due to the exploratory design and limited sample size, only univariate analyses were performed.

Statistical significance was set at p < 0.05. Correlation strength was defined as weak (*ρ* = 0.30–0.49), moderate (*ρ* = 0.50–0.69), or strong (*ρ* ≥ 0.70). Statistical analyses were performed using Microsoft Excel (Excel 2024) and GraphPad Prism (Prism 10.6.0).

## Results:

**Fig. 1:**
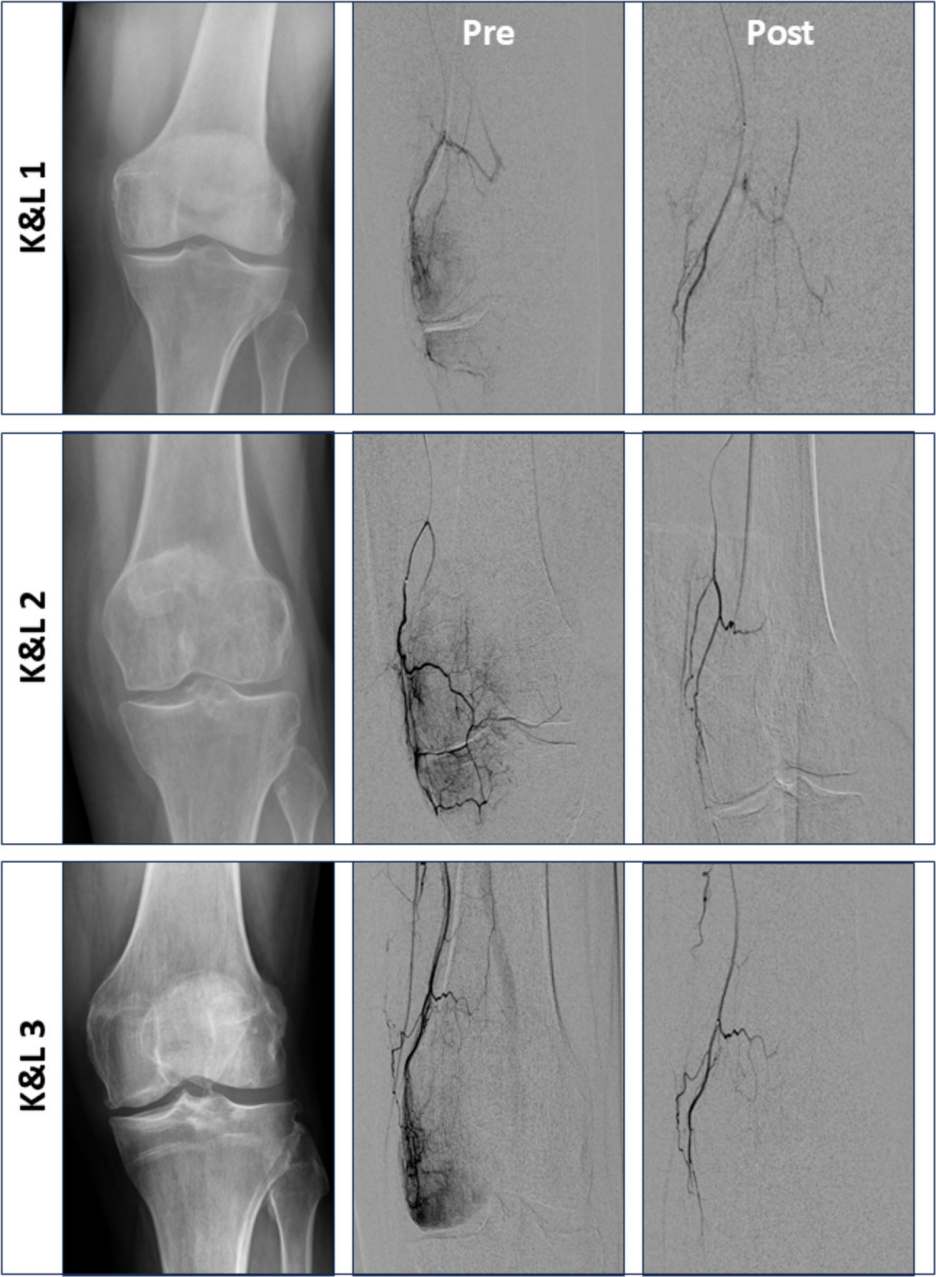

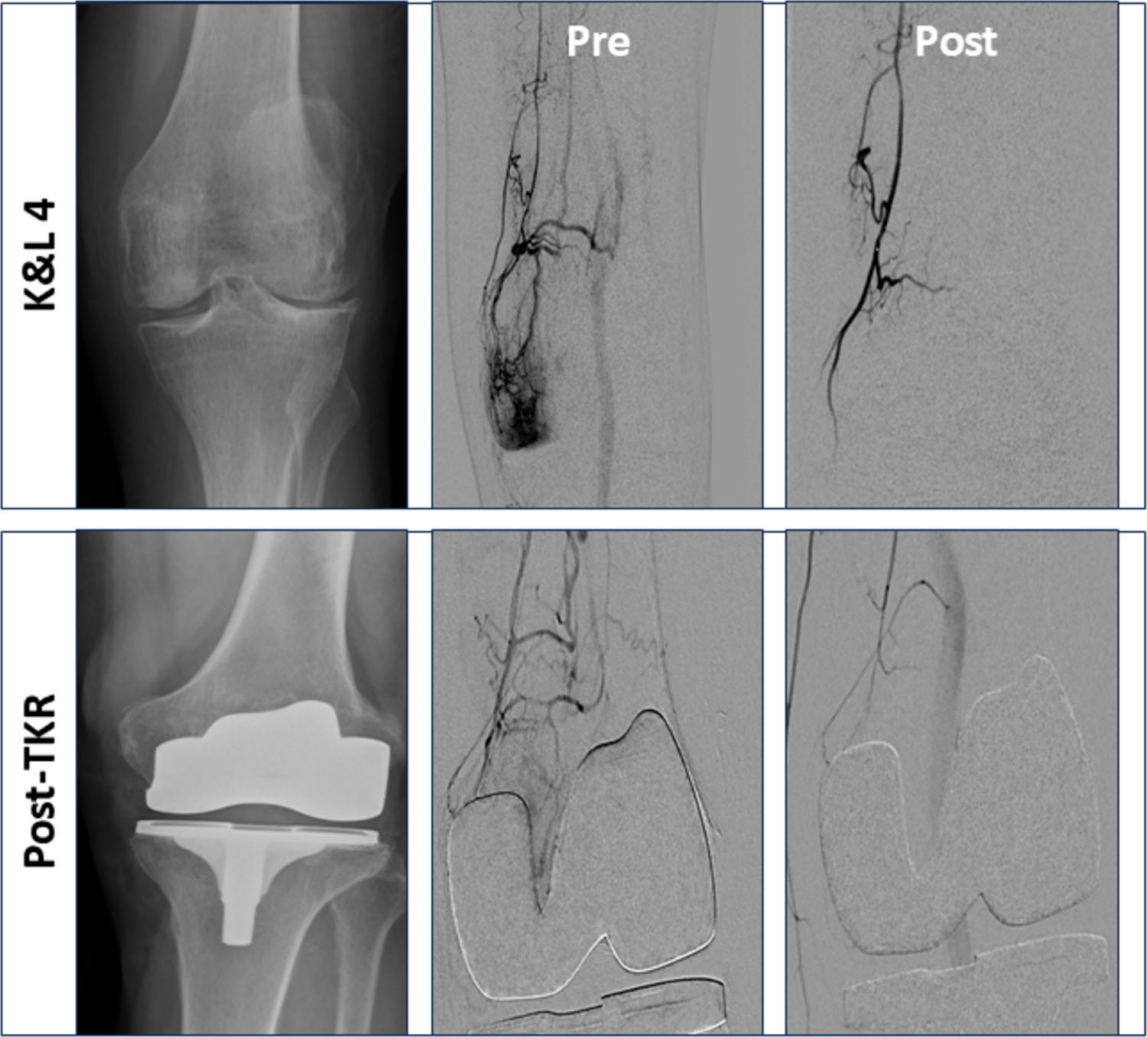
Embolization of the Descending Genicular Artery in different osteoarthritis grades DSA of the descending genicular artery in patients with different osteoarthritis grades and post-total knee replacement (post-TKR) before (pre) and after embolization (post). Embolization was performed using resorbable gelatin microspheres (RGM; Nexsphere, Next Biomedical, South Korea) diluted in 10 mL of iodinated contrast medium (300 mg/mL Accupaque, GE Healthcare, USA). The administered embolic volumes were 1.6 mL in Kellgren and Lawrence (K&L) grade 1, 2.1 mL in K&L grade 2, 2.6 mL in K&L grade 3, 2.9 mL in K&L grade 4, and 3.4 mL in post-TKR pain. In all patients, the embolization endpoint was defined as pruning, meaning disappearance of pathological hyperemic neovessels while maintaining inflow of the parent vessel

**Fig. 2:**
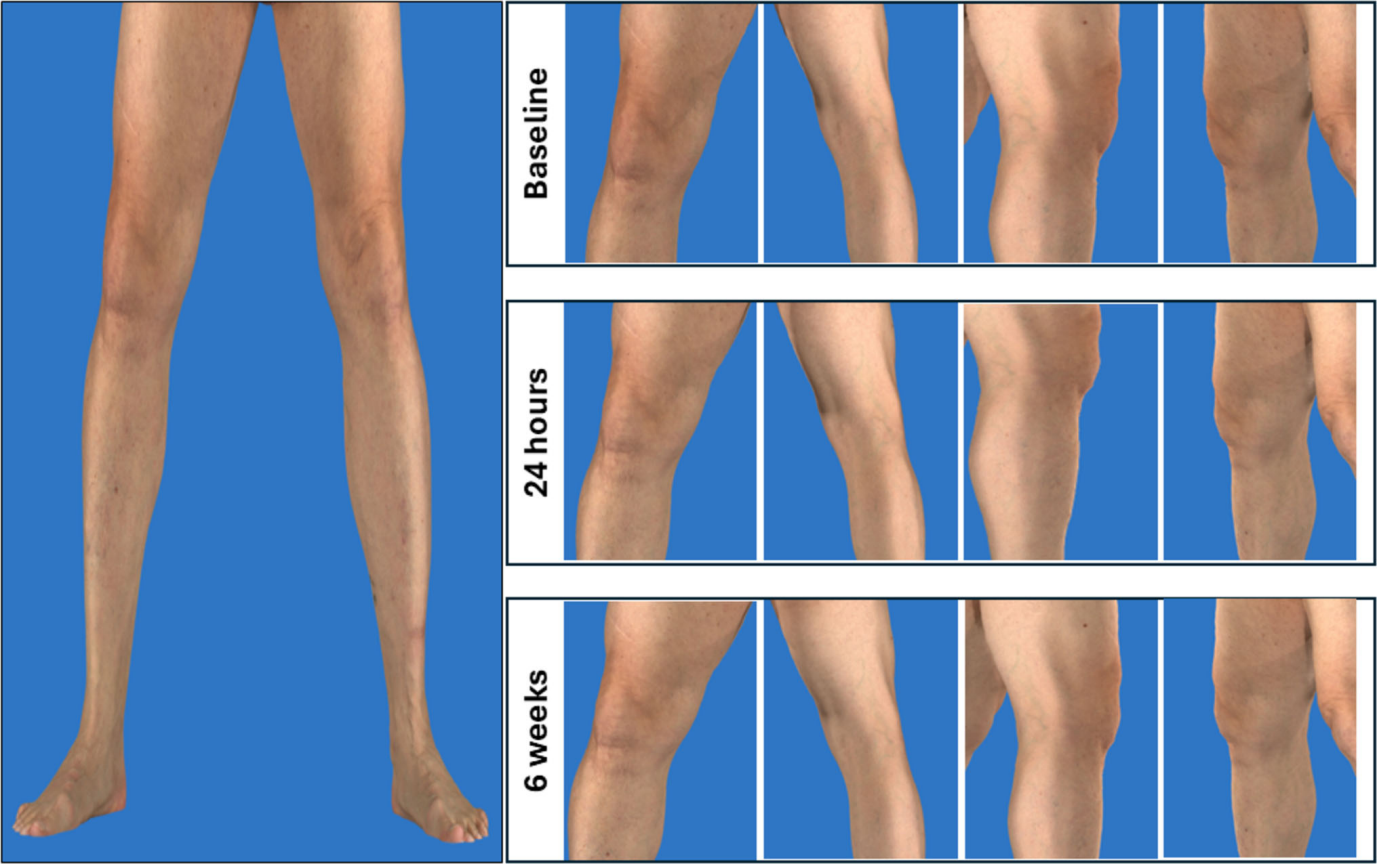
3D High-Resolution assessment of skin discolorations in Genicular Artery Embolization 3D high-resolution skin imaging (Vectra WB360, Canfield Scientific, USA) performed before, 24 h and 6 weeks after treatment. All images were reviewed by a board-certified dermatologist. No skin discolorations were observed at any time point

A total of 45 patients were treated. Table 1 summarizes patient characteristics and changes in pain treatments after GAE. Technical and clinical success was achieved in all patients. Figure 1 illustrates embolization of the descending genicular artery in patients with different OA grades as well as post-TKR. No skin discoloration or other complications were observed (Fig. 2). Table 2 provides an overview of procedural parameters.

**Table 1:** Patient baseline and follow-up characteristics

Age (years) median (range)	64 (28–82)	
Female, n (%)	24 (53%)	
BMI, median (range)	27 (19–41)	
Pain duration (years) median (range)	10 (1–40)	
Analgesia, n (%)	Baseline	6 months
Herbal medicines	16 (36%)	16 (36%)
NSAIDs	36 (80%)	25 (56%)
Opioids	9 (20%)	5 (11%)
Other pain treatments, n (%)	Baseline	6 months
Physiotherapy	34 (76%)	29 (64%)
Intra-articular injections	12 (27%)	0
PRP Injection	2 (4%)	0
*HADS Score*		
Anxiety Subscale		
Median (range)	6 (0–18)	
< 8 Points, n (%)	27 (60%)	
> 8 Points, n (%)	18 (40%)	
Depression Subscale		
Median (range)	6 (0–19)	
< 8 Points, n (%)	32 (71%)	
> 8 Points, n (%)	13 (29%)	
Side, n (%)		
Right	22 (49%)	
Left	23 (51%)	
OA severity (Kellgren–Lawrence Grade), n (%)		
1	5 (11%)	
2	14 (31%)	
3	10 (22%)	
4	6 (13%)	
Post-TKR	10 (22%)	

BMI: Body mass index, NSAIDs: Non-steroidal anti-inflammatory drugs, PRP: Platelet-rich plasma; HADS: Hospital Anxiety and Depression Scale, OA: Osteoarthritis, TKR: Total knee replacement

**Table 2:** Technical Procedural Parameters

Fluoroscopy time (minutes), mean (SD)	23 ± 7
Cumulative air kerma (mGy), mean (SD)	86 ± 129
Arteries embolized, n (%)	
Descending genicular artery	32 (71%)
Superomedial genicular artery	20 (44%)
Inferomedial genicular artery	34 (76%)
Superolateral genicular artery	28 (62%)
Inferolateral genicular artery	31 (69%)
Anterior recurrent tibial artery	10 (22%)
*Number of arteries embolized*	
median (range)	3 (2–6)
2, n (%)	6 (13%)
3, n (%)	19 (42%)
4, n (%)	15 (33%)
5, n (%)	4 (9%)
6, n (%)	1 (2%)
*Embolic volume (mL), median (range)*	
Total	6.5 (2.1–18.0)
Descending genicular artery	2.5 (0.5–6.0)
Superomedial genicular artery	1.8 (0.4–5.0)
Inferomedial genicular artery	2.0 (0.5–6.0)
Superolateral genicular artery	1.5 (0.5–6.0)
Inferolateral genicular artery	2.0 (0.3–5.0)
Anterior recurrent tibial artery	1.4 (0.6–4.0)

Baseline and follow-up assessments were complete for all 45 patients. KOOS and NRS scores were normally distributed. Both overall and within subgroups, there was a significant increase in all KOOS subscales and a decrease in NRS at every follow-up compared to baseline (Supplement 1–3, Table 3). No significant differences in KOOS change from baseline to six months were observed between OA grades or post-TKR. The median return-to-sport time was 5 days (1–8), and the median return-to-work time was 3 days (1–14).

**Table 3:** Outcome overall Mean scores in KOOS subscales and NRS at baseline and follow-up

Assessment	Visit	Mean ± SD	*p* value
KOOS: Pain	Baseline	42.1 ± 17.3	
	6 weeks	54.8 ± 21.4	< 0.0001
	3 months	58.3 ± 20.9	< 0.0001
	6 months	65.2 ± 22.2	< 0.0001
KOOS: Symptoms and stiffness	Baseline	46.6 ± 23.0	
	6 weeks	58.4 ± 26.1	< 0.01
	3 months	57.6 ± 23.2	< 0.01
	6 months	63.6 ± 22.8	< 0.01
KOOS: Daily living	Baseline	51.5 ± 23.7	
	6 weeks	61.0 ± 24.0	< 0.001
	3 months	65.3 ± 21.0	< 0.0001
	6 months	73.4 ± 23.1	< 0.001
KOOS: Sports and recreational activities	Baseline	19.2 ± 17.7	
	6 weeks	32.0 ± 29.1	< 0.01
	3 months	29.9 ± 26.1	< 0.01
	6 months	44.5 ± 27.7	< 0.001
KOOS: Quality of life	Baseline	23.2 ± 17.8	
	6 weeks	33.2 ± 23.4	< 0.001
	3 months	33.4 ± 21.9	< 0.01
	6 months	45.4 ± 22.8	< 0.0001
Numeric rating scale	Baseline	6.6 ± 1.4	
	6 weeks	4.9 ± 2.3	< 0.0001
	3 months	4.7 ± 2.2	< 0.0001
	6 months	4.4 ± 2.6	< 0.001

KOOS: Knee Injury and Osteoarthritis Outcome Score, NRS: Numeric Rating Scale

**Fig. 3:**
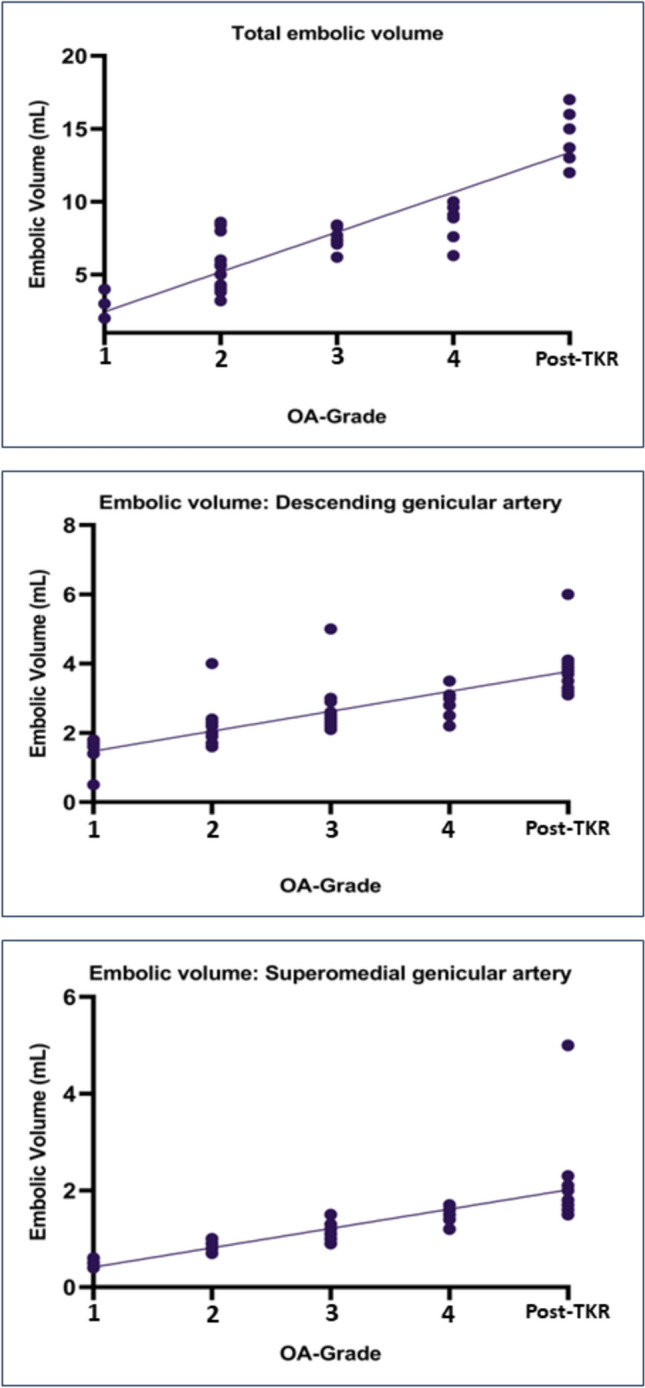

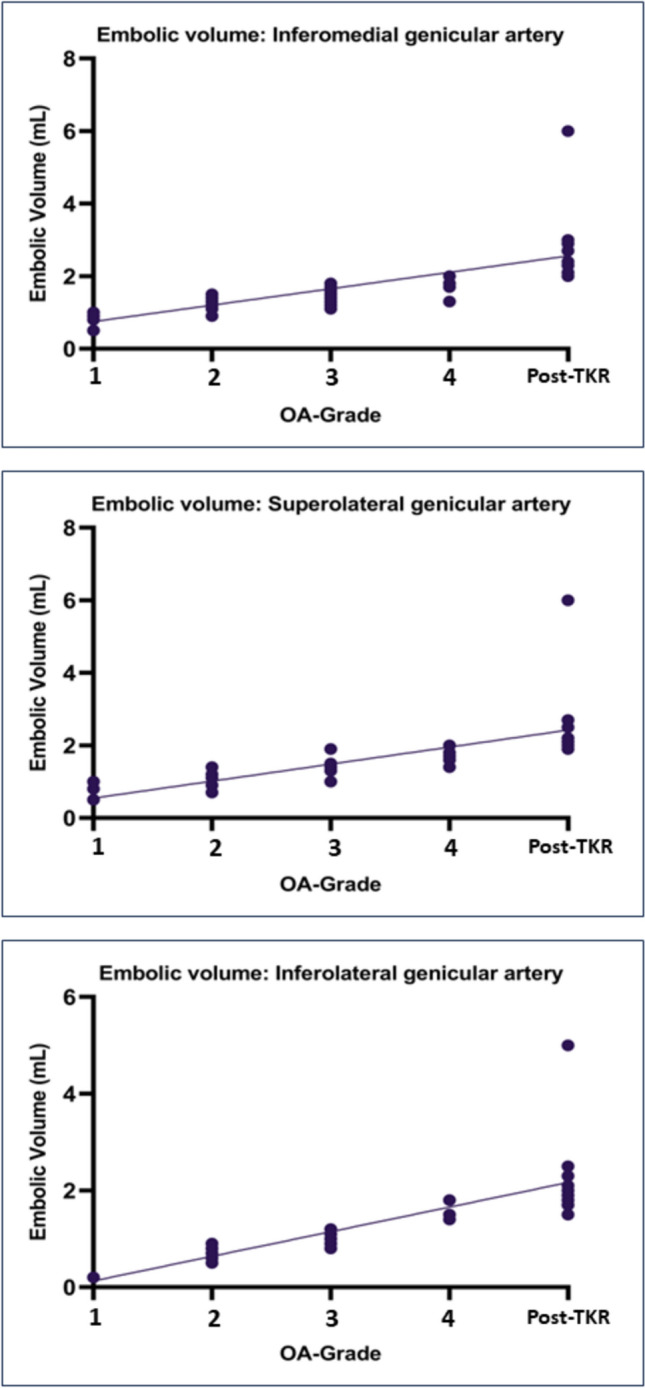

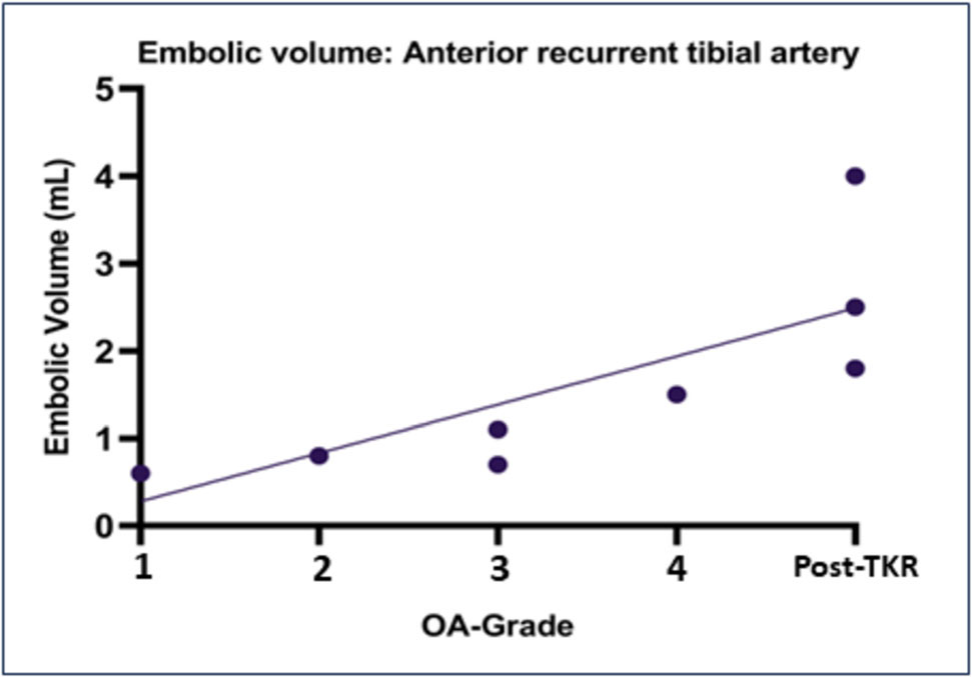
Linear regression of embolic volume in different osteoarthritis grades Linear regression between total and per-artery embolic volume versus different osteoarthritis grades (Kellgren and Lawrence Grades; K&L) and persistent pain after total knee replacement (post-TKR)

HADS scores, KOOS changes from baseline to six months and embolic volume were not normally distributed. Total and per-artery embolic volume differed significantly between OA grades and post-TKR (Supplement 4). Regression analyses demonstrated a significant positive correlation of both total and per-artery embolic volume with OA grade and post-TKR status (Fig. 3, Supplement 5).

Patients with HADS subscale score (anxiety/depression) ≥ 8 showed significantly smaller KOOS improvements and NRS reductions from baseline to six months compared with patients with HADS scores < 8. Regression analyses demonstrated significant negative correlations between HADS scores and improvement in KOOS and NRS for both HADS subscales (Supplement 6, Table 4) with moderate to weak correlation strengths. No significant correlation was observed between BMI and clinical outcome.

**Table 4:** Regression analyses and Spearman’s correlation of clinical outcome versus HADS score Linear regression and correlation analyses between improvement in KOOS subscales and NRS versus HADS subscale scores

		Improvement Baseline vs. 6 months, median (range)		β – Slope (95% CI)	*p* value	R^2^	Spearman’s ρ (95% CI)
		HADS < 8	HADS ≤ 8				
HADS-A	KOOS: Pain	19.5 (9.7–50)	9.8 (5.5–16.8)	- 0.8 [(−1.5) – (−0.1)]	< 0.05	0.05	0.17 (0.05–0.37)
	KOOS: Symptoms and Stiffness	20.8 (5.5–60.8)	3.8 (3.5–50)	- 2.2 [(−3.3) – (−1.0)]	< 0.001	0.21	0.43 (0.18–0.63)
	KOOS: Daily living	30.0 (8.8–61.8)	11.9 (1.5–48.5)	- 2.1 [(−3.3) – (−1.1)]	< 0.001	0.23	0.44 (0.19–0.64)
	KOOS: Sports and recreation	15.0 (5.0–95)	11.3 (0.0–25)	- 1.6 [(−3.0) – (−0.3)]	< 0.05	0.12	0.41 (0.13–0.64)
	KOOS: Quality of life	12.5 (5.5–93.8)	6.3 (0.0–31.3)	- 1.8 [(−3.2) – (−0.4)]	< 0.05	0.12	0.36 (0.09–0.57)
	Numeric rating scale	3.0 (1.0–6.0)	1.0 (0.0–5.0)	- 0.1 [(−0.3) – (−0.2)]	< 0.05	0.07	0.30 (0.09–0.48)
HADS-D	KOOS: Pain	33.3 (11.0–69.5)	22.1 (0.0–36.0)	- 1.1 [(−2.2) – (−0.1)]	< 0.05	0.10	0.20 (0.11–0.47)
	KOOS: Symptoms and Stiffness	32.3 (3.5–100)	21.5 (0.0–35.8)	- 0.6 [(−1.2) – (−0.1)]	< 0.05	0.02	0.08 (0.06–0.21)
	KOOS: Daily living	29.5 (5.7–78.3)	20.5 (0.0–32.0)	- 1.7 [(−2.8) – (−0.6)]	< 0.01	0.15	0.29 (0.02–0.52)
	KOOS: Sports and recreation	25.0 (5.0–100.0)	10.0 (3.0–27.0)	- 1.3 [(−2.3) – (−0.2)]	< 0.05	0.06	0.23 (0.01–0.42)
	KOOS: Quality of life	20.9 (6.3–68.8)	8.6 (3.0–31.3)	- 0.9 [(−1.7) – (−0.2)]	< 0.05	0.04	0.23 (0.06–0.39)
	Numeric rating scale	7.0 (1.0–9.0)	3.0 (1.0–8.0)	- 0.2 [(−0.3) – (−0.1)]	< 0.001	0.21	0.42 (0.23–0.58)

KOOS: Knee injury and Osteoarthritis Outcome Score; NRS: Numeric Ratings Scale; HADS-A: Hospital Anxiety and Depression Scale – subscale Anxiety; HADS-D: Hospital Anxiety and Depression Scale – subscale Depression

## Discussion:

This study demonstrates that GAE using RGM is safe and provides short-term clinical efficacy across all OA grades and in patients with post-TKR pain.

Clinical improvements continued between three and six months, consistent with prior studies [4, 5]. This suggests that gradual rather than immediate regression of synovial hypervascularization after GAE leads to progressive pain relief. Moreover, decreasing pain levels encourage healthier behavior in patients, reflected by reduced analgesic use in our cohort, further supporting continued benefit.

Clinical efficacy of GAE for mild to severe OA using permanent microspheres has already been demonstrated in several studies [15, 16, 23]. In contrast, data on temporary embolic agents in severe OA remain highly heterogeneous. Two studies have reported clinical failure in patients with severe OA after GAE with gelatin sponge particles and IMP/CS, with recurrence of pain after 3 months. In our study, however, patients with severe OA demonstrated sustained benefit up to six months. This discrepancy may be explained by the higher number of embolized vessels and greater embolic volume in our cohort, as studies have shown that incomplete treatment of genicular arteries yields no significant benefit over placebo [24]. With a mean of 6.5 mL the total embolic volume in our study is more than three times higher than the “common” volume defined by the Society of Interventional Radiology [25]. It was also substantially greater than in studies using RGM or other temporary embolics, despite comparable or even more aggressive embolization endpoints [4, 26, 27]. In line with our findings, Bhatia et al. treated a comparable number of arteries and applied similar volumes of IMP/CS in a smaller cohort with severe OA, achieving sustained clinical efficacy for up to 2 years without significant differences compared to permanent embolics [7].

In our study, the highest embolic volumes were applied in patients with post-TKR pain, with a median of 14.4 mL. Chau et al. reported comparable clinical outcomes using lower amounts of permanent microspheres [28]. In a retrospective analysis, four patients with post-TKR pain were treated with smaller volumes of RGM. However, the outcome was limited to the NRS and no subgroup analyses were performed. At present, the available data remain insufficient to provide firm recommendations regarding embolic agent selection and embolic volume for varying OA grades [8]. However, our findings add to the evolving evidence that different grades of OA may require distinct embolization strategies to achieve clinical success, underscoring the need for individualized, patient-adapted embolization strategies. The linear correlation between embolic volume and OA grade suggests that increasing OA severity may be accompanied by stronger synovitis and its angiographic correlate, the vascular blush, necessitating higher embolic volumes for effective treatment.

The interplay between pain and psychological comorbidities is complex, with both phenomena influencing each other. Patients with chronic pain are predisposed to anxiety and depression, while these comorbidities have also been identified as prognostic factors for reduced outcomes after treatment of OA pain [29, 30]. Thus, anxiety and depression may act as both amplifiers and consequences of pain. In our cohort, the proportion of patients with pathological HADS scores for depression and anxiety was higher than in the general population, reflecting the increased prevalence of these comorbidities in individuals with OA pain [31]. Elevated HADS scores have further been associated with greater pain catastrophizing [32]. Harrison et al. reported that patients with high levels of pain catastrophizing may benefit most from GAE [33]. In contrast, in our cohort, patients with elevated HADS scores showed less improvement after GAE compared with those with normal HADS values. This discrepancy may reflect methodological differences, as Harrison et al. assessed pain catastrophizing directly, whereas we used HADS, which captures broader psychological distress. Moreover, our cohort included more advanced OA and post-TKR patients, where structural damage may dominate psychological modulation of outcomes.

The observed changes in KOOS improvement per one-point increase in HADS ranged from 0.6 to 1.8 points, indicating that patients would need to increase by at least six points in HADS to reach a clinically relevant reduction in GAE outcome. This threshold is close to the established cutoff of eight points defining pathological HADS scores and is consistent with findings from conservative and surgical treatments for OA pain, where pathological HADS scores were associated with poorer outcomes [29, 30, 34]. The weak correlation coefficients and the discrepancy with the findings of Harrison et al. underline the multifactorial interplay between anxiety, depression and pain perception. However, our data underscore the importance of psychological screening as part of the pre-interventional workup prior to GAE, highlighting the need for a multidimensional approach in which both psychological assessment and GAE serve as complementary components of non-surgical pain management.

This study has several limitations. First, the sample size was relatively small, and the number of patients in each subgroup was limited, particularly those with K&L 4. However, significant improvements were observed even in this subgroup, with outcomes comparable to milder OA grades, suggesting a consistent treatment effect. As this pilot study was designed primarily to identify potential associations, only univariate analyses were performed. To the best of our knowledge, this is the first prospective study to evaluate RGM in GAE across different OA grades and post-TKR, underscoring the exploratory nature of our findings and the need for confirmation in larger cohorts.

Second, the IR was aware of the patients’ OA grades, which may have introduced observer bias in the choice of embolic volume. Yet, in real-world practice, IRs act as clinical partners and treat not only the angiographic blush but also the patient as a whole. Thus patient-specific considerations naturally contribute to treatment decisions.

Third, only short-term outcomes were assessed, limiting conclusions regarding long-term efficacy. However, the consistent clinical improvements observed up to six months provide a strong rationale for extended follow-up.

Another limitation, in addition to the absence of a control group, is that the embolization endpoint—although similar to that used in other studies—was defined subjectively based on the visual impression of the IR. To mitigate this, all procedures were performed by the same IR, with consistent preservation of parent vessel flow. However, the observed correlations between OA severity and embolic volume must be considered exploratory and definitive conclusions regarding the required embolic volume cannot yet be drawn.

Finally, no systematic imaging follow-up (e.g., MRI or ultrasound) was performed. This was a deliberate choice to concentrate on clinical efficacy and safety, which are most relevant for patients.

In conclusion, GAE using RGM is safe and provides short-term clinical success across all OA grades as well as in post-TKR pain. Patients with higher OA severity received larger embolic volumes, which appear safe when temporary embolics such as RGM are used. Anxiety and depression were associated with reduced clinical benefit, underscoring the importance of psychological screening as part of the pre-procedural assessment. Overall, our findings emphasize that patient-centered thinking should not stop at the doors of the angio suite: Embolization strategies need to be tailored to individual patient needs and integrated as part of a comprehensive, multidisciplinary approach to pain management.

## Supplementary Information

Below is the link to the electronic supplementary material.

Supplementary file1 (PNG 61 KB)

Supplementary file2 (PNG 62 KB)

Supplementary file3 (PNG 160 KB)

Supplementary file4 (PNG 110 KB)

Supplementary file5 (DOCX 26 KB)

Supplementary file6 (DOCX 15 KB)

Supplementary file7 (DOCX 15 KB)

Supplementary file8 (PNG 137 KB)
