# Supplementary figures and images for: Genicular Artery Embolization Using Resorbable Gelatin Microspheres for Refractory Knee Pain: Technique, Safety and Clinical Outcome
Source: Cardiovasc Intervent Radiol. 2025 Nov 18;49(2):356–67. doi: 10.1007/s00270-025-04274-6 (PMC12868021; doi:10.1007/s00270-025-04274-6)

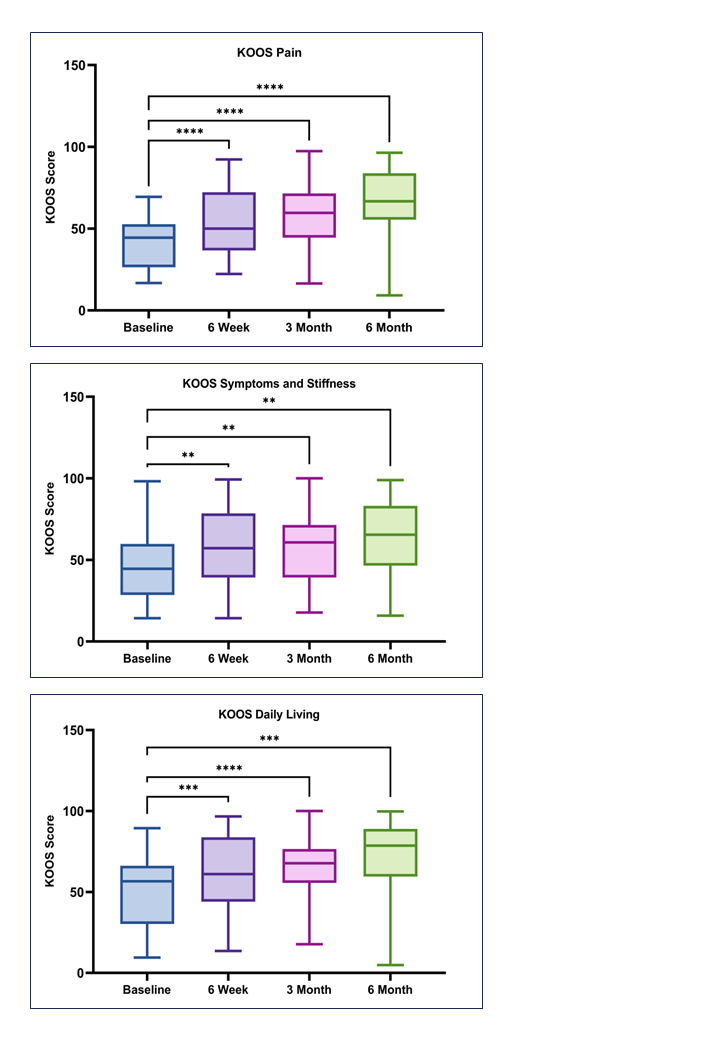

Supplement: Supplementary file 1 — Supplementary file1 (PNG 61 KB) [file 270_2025_4274_MOESM1_ESM.png]

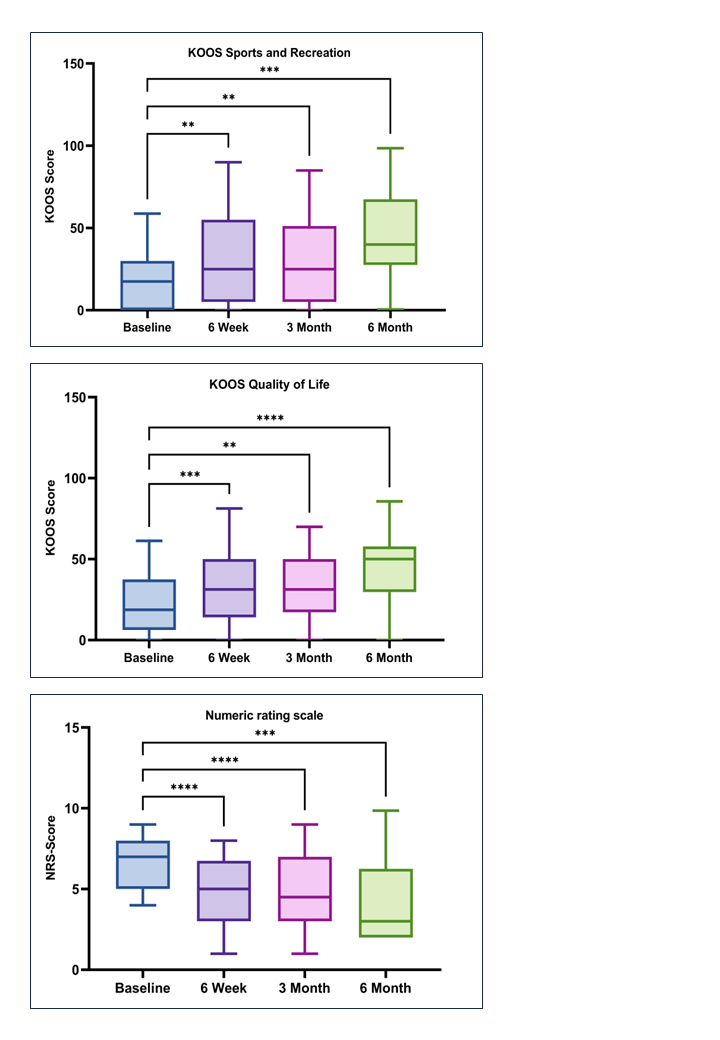

Supplement: Supplementary file 2 — Supplementary file2 (PNG 62 KB) [file 270_2025_4274_MOESM2_ESM.png]

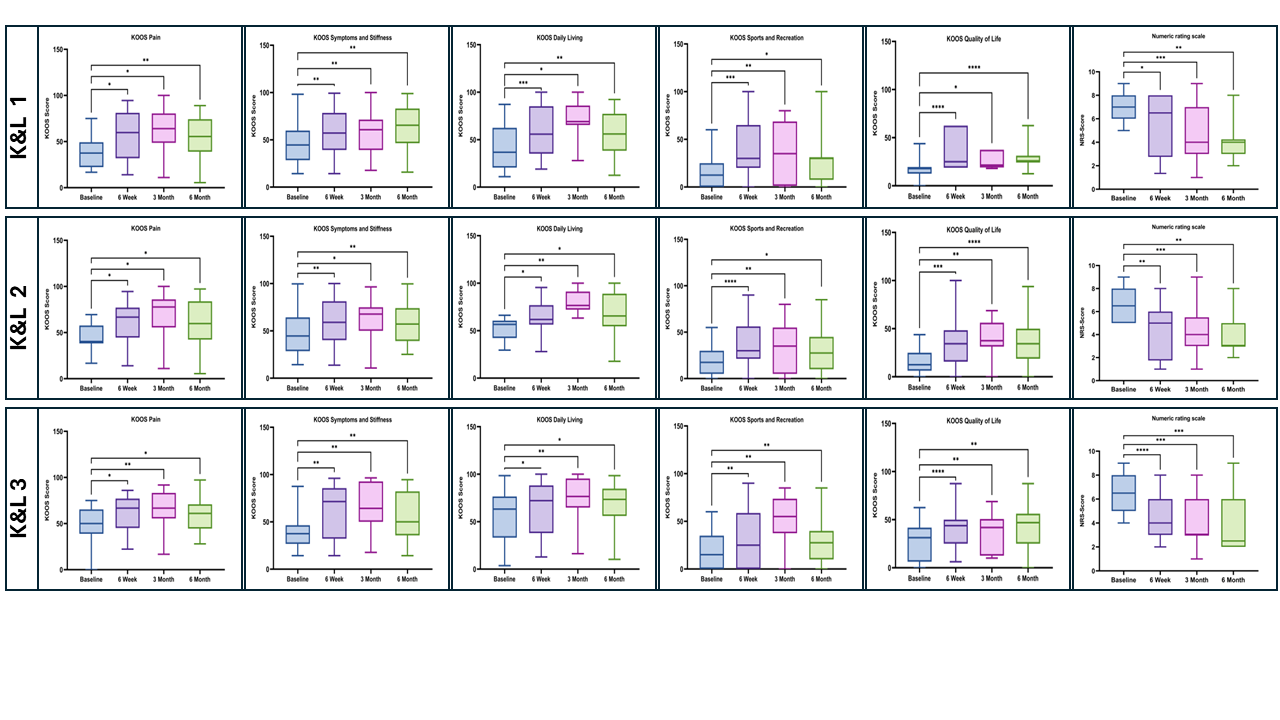

Supplement: Supplementary file 3 — Supplementary file3 (PNG 160 KB) [file 270_2025_4274_MOESM3_ESM.png]

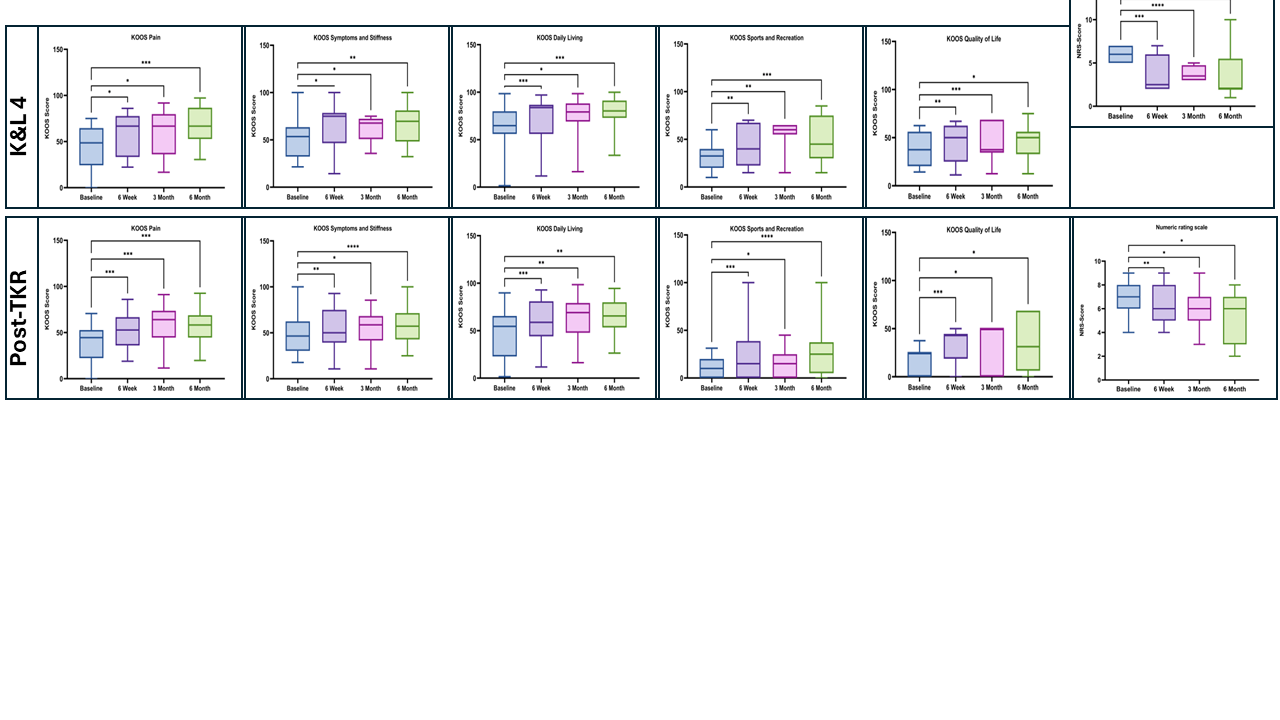

Supplement: Supplementary file 4 — Supplementary file4 (PNG 110 KB) [file 270_2025_4274_MOESM4_ESM.png]

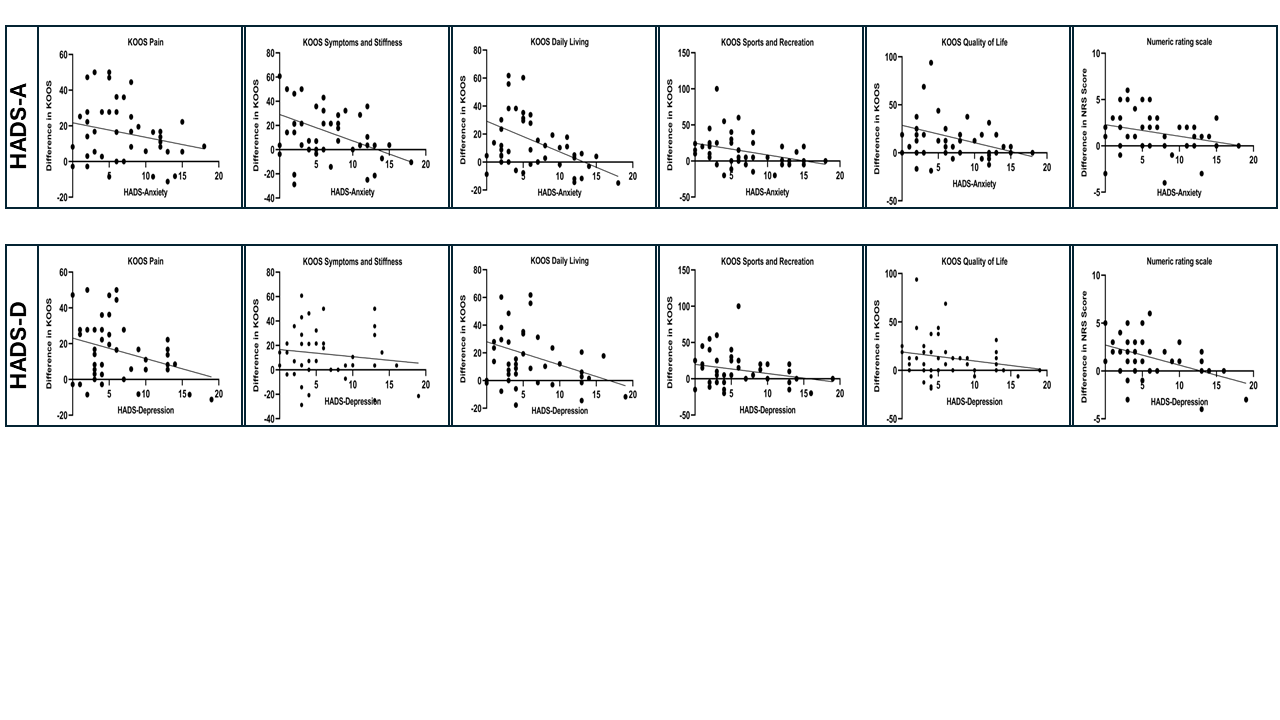

Supplement: Supplementary file 8 — Supplementary file8 (PNG 137 KB) [file 270_2025_4274_MOESM8_ESM.png]
