# Supplementary material for: Genicular Artery Embolization Using Resorbable Gelatin Microspheres for Refractory Knee Pain: Technique, Safety and Clinical Outcome
Source: Cardiovasc Intervent Radiol. 2025 Nov 18;49(2):356–67. doi: 10.1007/s00270-025-04274-6 (PMC12868021; doi:10.1007/s00270-025-04274-6)
Supplement: Supplementary file 5 — Supplementary file5 (DOCX 26 KB) [file 270_2025_4274_MOESM5_ESM.docx]

|  |  | **Visit** | **Mean ± SD** | ***p* value** |
| --- | --- | --- | --- | --- |
| **KOOS: Pain** | **K&L 1** | Baseline | 38.4 ± 18.0 |  |
|  |  | 6 weeks | 56.7 ± 26.7 | < 0.05 |
|  |  | 3 months | 62.7 ± 25.6 | < 0.05 |
|  |  | 6 months | 54.0 ± 22.7 | < 0.01 |
|  | **K&L 2** | Baseline | 44.5 ± 15.1 |  |
|  |  | 6 weeks | 60.9 ± 24.5 | < 0.05 |
|  |  | 3 months | 68.6 ± 29.1 | < 0.05 |
|  |  | 6 months | 59.7 ± 25.9 | < 0.05 |
|  | **K&L 3** | Baseline | 48.2 ± 19.1 |  |
|  |  | 6 weeks | 61.3 ± 20.1 | < 0.05 |
|  |  | 3 months | 65.2 ± 21.5 | < 0.01 |
|  |  | 6 months | 61.2 ± 19.1 | < 0.05 |
|  | **K&L 4** | Baseline | 44.2 ± 22.1 |  |
|  |  | 6 weeks | 57.5 ± 23.0 | < 0.05 |
|  |  | 3 months | 60.0 ± 26.4 | < 0.05 |
|  |  | 6 months | 66.9 ± 21.3 | < 0.001 |
|  | **post-TKR** | Baseline | 41.2 ± 19.7 |  |
|  |  | 6 weeks | 52.9 ± 20.3 | < 0.001 |
|  |  | 3 months | 56.9 ± 23.9 | < 0.001 |
|  |  | 6 months | 56.3 ± 20.0 | < 0.001 |
| **KOOS: Symptoms and stiffness** | **K&L 1** | Baseline | 43.4 ± 22.5 |  |
|  |  | 6 weeks | 59.2 ± 29.0 | < 0.01 |
|  |  | 3 months | 57.0 ± 23.4 | < 0.05 |
|  |  | 6 months | 56.4 ± 23.8 | < 0.05 |
|  | **K&L 2** | Baseline | 48.8 ± 24.4 |  |
|  |  | 6 weeks | 60.0 ± 26.0 | < 0.01 |
|  |  | 3 months | 62.6 ± 21.2 | < 0.05 |
|  |  | 6 months | 58.0 ± 22.8 | < 0.01 |
|  | **K&L 3** | Baseline | 40.5 ± 20.4 |  |
|  |  | 6 weeks | 58.9 ± 29.7 | < 0.01 |
|  |  | 3 months | 61.0 ± 27.3 | < 0.01 |
|  |  | 6 months | 56.2 ± 24.8 | < 0.01 |
|  | **K&L 4** | Baseline | 52.2 ± 20.1 |  |
|  |  | 6 weeks | 67.0 ± 23.0 | < 0.05 |
|  |  | 3 months | 61.8 ± 13.6 | < 0.05 |
|  |  | 6 months | 67.6 ± 20.8 | < 0.01 |
|  | **post-TKR** | Baseline | 48.7 ± 25.2 |  |
|  |  | 6 weeks | 54.8 ± 25.7 | < 0.01 |
|  |  | 3 months | 54.3 ± 25.3 | < 0.05 |
|  |  | 6 months | 59.5 ± 22.4 | < 0.0001 |

|  |  | **Visit** | **Mean ± SD** | ***p* value** |
| --- | --- | --- | --- | --- |
| **KOOS: Daily living** | **K&L 1** | Baseline | 42.9 ± 25.0 |  |
|  |  | 6 weeks | 60.2 ± 25.4 | < 0.001 |
|  |  | 3 months | 71.5 ± 20.7 | < 0.05 |
|  |  | 6 months | 55.0 ± 24.5 | < 0.01 |
|  | **K&L 2** | Baseline | 52.3 ± 11.9 |  |
|  |  | 6 weeks | 64.4 ± 19.3 | < 0.05 |
|  |  | 3 months | 79.9 ± 12.6 | < 0.01 |
|  |  | 6 months | 66.9 ± 22.4 | < 0.05 |
|  | **K&L 3** | Baseline | 55.8 ± 28.5 |  |
|  |  | 6 weeks | 65.5 ± 29.5 | < 0.05 |
|  |  | 3 months | 72.5 ± 26.0 | < 0.01 |
|  |  | 6 months | 67.1 ± 25.6 | < 0.05 |
|  | **K&L 4** | Baseline | 61.0 ± 27.3 |  |
|  |  | 6 weeks | 71.3 ± 25.6 | < 0.001 |
|  |  | 3 months | 71.9 ± 26.4 | < 0.05 |
|  |  | 6 months | 79.3 ± 16.4 | < 0.001 |
|  | **post-TKR** | Baseline | 47.4 ± 26.1 |  |
|  |  | 6 weeks | 59.8 ± 22.1 | < 0.001 |
|  |  | 3 months | 63.7 ± 24.5 | < 0.01 |
|  |  | 6 months | 64.3 ± 20.2 | < 0.01 |
| **KOOS: Sports and recreational activities** | **K&L 1** | Baseline | 16.2 ± 17.8 |  |
|  |  | 6 weeks | 39.7 ± 30.8 | < 0.001 |
|  |  | 3 months | 35.0 ± 30.5 | < 0.01 |
|  |  | 6 months | 30.6 ± 30.0 | < 0.05 |
|  | **K&L 2** | Baseline | 18.6 ± 15.7 |  |
|  |  | 6 weeks | 38.8 ± 27.6 | < 0.0001 |
|  |  | 3 months | 36.4 ± 26.4 | < 0.01 |
|  |  | 6 months | 30.7 ± 26.9 | < 0.05 |
|  | **K&L 3** | Baseline | 19.5 ± 21.3 |  |
|  |  | 6 weeks | 31.9 ± 34.0 | < 0.01 |
|  |  | 3 months | 52.0 ± 30.3 | < 0.01 |
|  |  | 6 months | 30.0 ± 25.6 | < 0.01 |
|  | **K&L 4** | Baseline | 32.8 ± 17.4 |  |
|  |  | 6 weeks | 43.8 ± 22.4 | < 0.01 |
|  |  | 3 months | 55.0 ± 16.9 | < 0.01 |
|  |  | 6 months | 50.0 ± 26.7 | < 0.001 |
|  | **post-TKR** | Baseline | 11.5 ± 13.0 |  |
|  |  | 6 weeks | 26.4 ± 31.9 | < 0.001 |
|  |  | 3 months | 15.8 ± 15.7 | < 0.05 |
|  |  | 6 months | 30.4 ± 28.7 | < 0.0001 |

|  |  | **Visit** | **Mean ± SD** | ***p* value** |
| --- | --- | --- | --- | --- |
| **KOOS: Quality of life** | **K&L 1** | Baseline | 18.8 ± 14.8 |  |
|  |  | 6 weeks | 35.4 ± 20.5 | < 0.01 |
|  |  | 3 months | 25.8 ± 8.8 | < 0.05 |
|  |  | 6 months | 31.2 ± 17.4 | < 0.0001 |
|  | **K&L 2** | Baseline | 16.8 ± 13.1 |  |
|  |  | 6 weeks | 35.4 ± 25.4 | < 0.001 |
|  |  | 3 months | 40.2 ± 21.9 | < 0.01 |
|  |  | 6 months | 33.9 ± 25.5 | < 0.0001 |
|  | **K&L 3** | Baseline | 27.9 ± 19.8 |  |
|  |  | 6 weeks | 38.9 ± 24.5 | < 0.0001 |
|  |  | 3 months | 38.1 ± 21.3 | < 0.01 |
|  |  | 6 months | 40.0 ± 25.3 | < 0.01 |
|  | **K&L 4** | Baseline | 37.3 ± 17.4 |  |
|  |  | 6 weeks | 42.7 ± 18.6 | < 0.01 |
|  |  | 3 months | 46.2 ± 19.8 | < 0.001 |
|  |  | 6 months | 44.7 ± 19.2 | < 0.05 |
|  | **post-TKR** | Baseline | 19.1 ± 12.4 |  |
|  |  | 6 weeks | 31.6 ± 17.6 | < 0.001 |
|  |  | 3 months | 31.8 ± 22.1 | < 0.05 |
|  |  | 6 months | 31.5 ± 24.8 | < 0.05 |
| **Numeric rating scale** | **K&L 1** | Baseline | 7.0 ± 1.4 |  |
|  |  | 6 weeks | 5.5 ± 2.6 | < 0.05 |
|  |  | 3 months | 4.8 ± 2.1 | < 0.001 |
|  |  | 6 months | 4.0 ± 1.6 | < 0.01 |
|  | **K&L 2** | Baseline | 6.6 ± 1.3 |  |
|  |  | 6 weeks | 4.4 ± 2.5 | < 0.01 |
|  |  | 3 months | 4.3 ± 2.3 | < 0.001 |
|  |  | 6 months | 4.0 ± 1.9 | < 0.01 |
|  | **K&L 3** | Baseline | 6.4 ± 1.6 |  |
|  |  | 6 weeks | 4.5 ± 2.3 | < 0.0001 |
|  |  | 3 months | 4.1 ± 2.3 | < 0.001 |
|  |  | 6 months | 4.0 ± 2.7 | < 0.001 |
|  | **K&L 4** | Baseline | 6.0 ± 1.0 |  |
|  |  | 6 weeks | 3.7 ± 2.2 | < 0.001 |
|  |  | 3 months | 3.8 ± 0.9 | < 0.0001 |
|  |  | 6 months | 3.7 ± 2.9 | < 0.05 |
|  | **post-TKR** | Baseline | 6.8 ± 1.4 |  |
|  |  | 6 weeks | 6.2 ± 1.6 | < 0.01 |
|  |  | 3 months | 6.1 ± 1.8 | < 0.05 |
|  |  | 6 months | 5.2 ± 2.4 | < 0.05 |
