# Supplementary material for: Genicular Artery Embolization Using Resorbable Gelatin Microspheres for Refractory Knee Pain: Technique, Safety and Clinical Outcome
Source: Cardiovasc Intervent Radiol. 2025 Nov 18;49(2):356–67. doi: 10.1007/s00270-025-04274-6 (PMC12868021; doi:10.1007/s00270-025-04274-6)
Supplement: Supplementary file 6 — Supplementary file6 (DOCX 15 KB) [file 270_2025_4274_MOESM6_ESM.docx]

|  | **K&L 1** | **K&L 2** | **K&L 3** | **K&L 4** | **Post-TKR** | ***p*-Value** |
| --- | --- | --- | --- | --- | --- | --- |
| **Total (mL)** | 3.0  (2.1-4.0) | 5.3  (3.2-8.6) | 7.4 (6.2-8.4) | 9.0  (6.3-10.0) | 14.4  (12.0-18.0) | < 0.0001 |
| **DGA (mL)** | 1.5  (0.5-1.9) | 2.0  (1.5-2.5) | 2.5 (1.9-3.1) | 2.8  (2.1-3.5) | 3.5  (2.6-6.0) | < 0.0001 |
| **SMGA (mL)** | 0.5  (0.4-0.6) | 0.8  (0.6-1.0) | 1.2 (0.9-1.5) | 1.5  (1.1-1.9) | 2.0  (1.5-5.0) | < 0.05 |
| **IMGA (mL)** | 1.0  (0.5-1.2) | 1.2  (0.9-1.5) | 1.5 (1.1-1.9) | 1.7  (1.3-2.1) | 2.5  (1.9-6.0) | < 0.05 |
| **SLGA (mL)** | 0.8  (0.5-1.0) | 1.1  (0.7-1.4) | 1.4 (1.3-1.9) | 1.8  (1.4-2.0) | 2.7  (2.1-6.0) | < 0.05 |
| **ILGA (mL)** | 0.3  (0.3-0.4) | 0.7  (0.5-0.9) | 1.0 (0.8-1.2) | 1.3  (1.0-1.6) | 2.0  (1.5-5.0) | < 0.001 |
| **ARTA (mL)** | 0.6  (0.3-0.8) | 0.8  (0.5-0.9) | 0.9  (0.7-1.1) | 1.5  (1.1-1.9) | 2.5  (1.8-4.0) | < 0.05 |
