# Supplementary material for: Genicular Artery Embolization Using Resorbable Gelatin Microspheres for Refractory Knee Pain: Technique, Safety and Clinical Outcome
Source: Cardiovasc Intervent Radiol. 2025 Nov 18;49(2):356–67. doi: 10.1007/s00270-025-04274-6 (PMC12868021; doi:10.1007/s00270-025-04274-6)
Supplement: Supplementary file 7 — Supplementary file7 (DOCX 15 KB) [file 270_2025_4274_MOESM7_ESM.docx]

| **Embolic volume vs. OA-Grade** | **β - Slope (95% CI)** | ***p* value** | **R^2^** | **Spearman’s ρ**  **(95% CI)** |
| --- | --- | --- | --- | --- |
| **Total** | 2.7 (2.3-3.1) | < 0.001 | 0.82 | 0.9 (0.81-0.94) |
| **DGA** | 0.6 (0.4-0.6) | < 0.01 | 0.56 | 0.82 (0.69-0.9) |
| **SMGA** | 0.4 (0.3-0.5) | < 0.01 | 0.54 | 0.94 (0.91-0.97) |
| **IMGA** | 0.5 (0.3-0.6) | < 0.01 | 0.51 | 0.86 (0.75-0.92) |
| **SLGA** | 0.5 (0.3-0.6) | < 0.01 | 0.52 | 0.91 (0.83-0.94) |
| **ILGA** | 0.5 (0.4-0.6) | < 0.01 | 0.67 | 0.95 (0.91-0.97) |
| **ARTA** | 0.6 (0.2-0.9) | < 0.05 | 0.65 | 0.93 (0.9-0.95) |
